# Early goal-directed resuscitation of patients with septic shock: current evidence and future directions

**DOI:** 10.1186/s13054-015-1011-9

**Published:** 2015-08-28

**Authors:** Ravi G. Gupta, Sarah M. Hartigan, Markos G. Kashiouris, Curtis N. Sessler, Gonzalo M. L. Bearman

**Affiliations:** Division of Pulmonary Disease and Critical Care Medicine, Department of Internal Medicine, Virginia Commonwealth University School of Medicine, P.O. Box 980050, Richmond, VA 23298 USA; Division of General Internal Medicine, Department of Internal Medicine, Virginia Commonwealth University School of Medicine, P.O. Box 980070, Richmond, VA 23298 USA; Division of Infectious Diseases, Department of Internal Medicine, Virginia Commonwealth University School of Medicine, P.O. Box 980019, Richmond, VA 23298 USA

## Abstract

Severe sepsis and septic shock are among the leading causes of mortality in the intensive care unit. Over a decade ago, early goal-directed therapy (EGDT) emerged as a novel approach for reducing sepsis mortality and was incorporated into guidelines published by the international Surviving Sepsis Campaign. In addition to requiring early detection of sepsis and prompt initiation of antibiotics, the EGDT protocol requires invasive patient monitoring to guide resuscitation with intravenous fluids, vasopressors, red cell transfusions, and inotropes. The effect of these measures on patient outcomes, however, remains controversial. Recently, three large randomized trials were undertaken to re-examine the effect of EGDT on morbidity and mortality: the ProCESS trial in the United States, the ARISE trial in Australia and New Zealand, and the ProMISe trial in England. These trials showed that EGDT did not significantly decrease mortality in patients with septic shock compared with usual care. In particular, whereas early administration of antibiotics appeared to increase survival, tailoring resuscitation to static measurements of central venous pressure and central venous oxygen saturation did not confer survival benefit to most patients. In the following review, we examine these findings as well as other evidence from recent randomized trials of goal-directed resuscitation. We also discuss future areas of research and emerging paradigms in sepsis trials.

## Introduction

The Italian philosopher Niccolo Machiavelli wrote in his classic treatise *The Prince*, ‘a hectic fever, in its beginning, is difficult to recognize but easy to cure; in the course of time, it becomes easy to recognize but difficult to cure’. Five centuries later, his insightful observation remains largely true for patients with sepsis. With over 750,000 cases documented in the United States each year, severe sepsis and septic shock are among the leading causes of mortality in critically ill patients and cost the health-care system nearly $17 billion annually [1, 2]. The incidence of sepsis has increased over the last two decades, a trend likely driven by aging patient populations, the emergence of drug-resistant pathogens, and increased use of immunosuppressive drugs [3, 4]. Mortality rates remain high and range from 10 % to 50 % despite advances in critical care medicine [5, 6].

Although our understanding of the pathophysiology of sepsis has significantly improved since the 1970s, current treatment options continue to focus primarily on antibiotics and supportive care. An influential study by Rivers and colleagues [7] in 2001 introduced a novel treatment protocol called early goal-directed therapy (EGDT), which was shown to increase survival among patients with septic shock at a single institution. The EGDT protocol comprised a set of tasks to be completed within the first 6 hours of presentation, including the placement of a central venous catheter to monitor hemodynamic variables during fluid resuscitation. Resuscitation was titrated to specific targets of central venous pressure (CVP), mean arterial pressure (MAP), and central venous oxygen saturation (ScvO_2_). In 2004, this approach was adopted by the international Surviving Sepsis Campaign (SSC) and incorporated into sepsis care ‘bundles’ that continue to be used in the intensive care unit (ICU) [8].

Although there is broad consensus that early diagnosis of sepsis and prompt initiation of antibiotics improve patient survival, methods of initial resuscitation and hemodynamic monitoring remain controversial [9, 10]. Previous studies have shown that CVP is a poor marker of fluid responsiveness in critically ill patients [11]. Furthermore, although central venous catheters may offer valuable information during resuscitation, they can result in complications such as pneumothorax and infection in over 15 % of patients [12]. The debate was recently revisited with the publication of randomized controlled trials of EGDT that failed to replicate positive findings of the original study by Rivers and colleagues. This new evidence may have significant implications for future iterations of SSC guidelines and clinical practice. In the following review, we discuss current evidence for early goal-directed resuscitation of patients with septic shock as well as novel areas of research.

## Pathophysiology of septic shock

The American College of Chest Physicians and Society of Critical Care Medicine have established clinical criteria for systemic inflammatory response syndrome, sepsis, severe sepsis, and septic shock (Table 1) [13]. The pathogenic sequence of sepsis begins with the growth of microorganisms at a site of infection, most commonly in the lungs, abdomen, or urinary tract. The infection either spreads into the bloodstream and results in positive blood cultures or grows locally and induces factors that stimulate the release of endogenous mediators of systemic inflammation [14]. These mediators can have a significant effect on the vasculature and heart, ultimately manifesting as hypotension, systemic hypoperfusion, and progressive failure of multiple organs.

**Table 1:** Diagnostic criteria for sepsis

Diagnosis	Clinical criteria
Systemic inflammatory response syndrome (SIRS)	Two or more of the following:
	- Fever (core temperature of more than 38 °C) or hypothermia (core temperature of less than 36 °C)
	- Heart rate of more than 90 beats per minute
	- Respiratory rate of more than 20 breaths per minute or partial pressure of carbon dioxide in arterial blood (PaCO_2_) of less than 32 mm Hg
	- Leukocytosis (white-cell count of more than 12,000 cells/μl) or leukopenia (white-cell count of less than 4,000 cells/μl) or more than 10 % immature forms (bands)
Sepsis	Confirmed infection and at least two SIRS criteria
Severe sepsis	Sepsis and organ dysfunction as evidenced by arterial hypoxemia, lactic acidosis, oliguria, altered mental status, and so on
Septic shock	Sepsis and hypotension refractory to fluid resuscitation

Hypotension in sepsis occurs due to peripheral vasodilation and redistribution of intravascular fluid. Vasodilation is thought to result from the release of vasoactive mediators produced by vascular endothelial cells, including prostacyclin and nitric oxide [15, 16]. Inflammatory cytokines disrupt endothelial cell junctions, causing increased capillary permeability and fluid shift into the extravascular space. The overall effect of these changes is tissue hypoperfusion and organ dysfunction, although cardiac output is usually preserved or increased. Occasionally, sepsis can depress the myocardium, causing refractory distributive and cardiogenic shock [17].

**Fig. 1:**
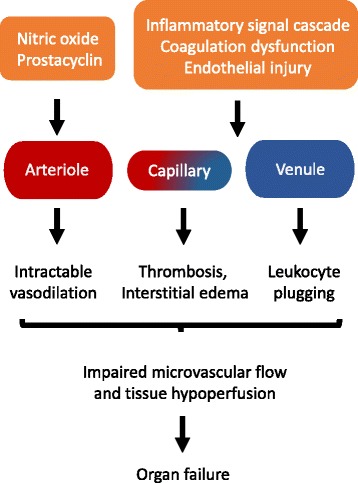
Microcirculatory dysfunction in sepsis. The microvascular network undergoes functional and structural changes during inflammatory states such as sepsis and may have a key role in organ dysfunction. Changes include dilation of arterioles, microvascular thrombosis, increased adhesion of leukocytes in venules, and increased vascular permeability. These alterations result in impaired microcirculatory blood flow and tissue perfusion, ultimately leading to organ failure. Techniques for measuring microcirculatory flow in vivo have been previously described but these tools have not yet been rigorously tested for use in patients with sepsis

A central goal in the treatment of septic shock, therefore, is the maintenance of adequate tissue perfusion with hemodynamic support, which includes intravenous fluid resuscitation and administration of vasopressors, inotropes, and packed red blood cells. This general approach is predicated upon the theory that organ dysfunction results from intravascular volume depletion, peripheral vasodilation, and myocardial depression. A growing body of evidence suggests that impairment of the microvascular network may also have key pathogenic roles (Fig. 1) [18–20]. However, few studies have examined the physiologic effects of intravenous fluid resuscitation beyond 30 to 60 minutes post-administration, and the effects of fluid bolus therapy on microcirculation remain poorly understood [21].

## Initial management

The SSC advocates early recognition of septic shock and initiation of empiric antibiotics within the first hour of treatment. The SSC also recommends obtaining at least two blood cultures prior to initiation of antibiotics. This recommendation is based on data from a retrospective study by Kumar and colleagues [22], which showed that each hour delay in the administration of appropriate antibiotics was associated with a 7.6 % increase in mortality. Other observational studies have provided similar evidence of the survival benefit conferred by appropriate antimicrobial therapy [23]. Despite a lack of evidence from prospective randomized trials, few experts would argue against the use of these measures to identify and control the source of infection in sepsis [24].

**Fig. 2:**
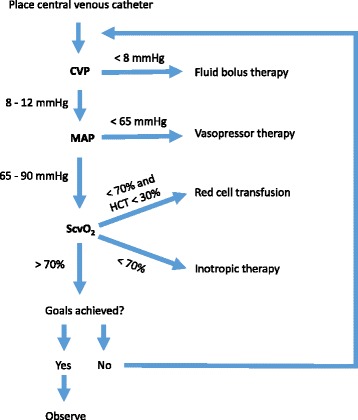
Early goal-directed therapy. During the first 6 hours of septic shock, the early goal-directed therapy protocol requires the placement of a central venous catheter with an oximetric port for continuous monitoring of central venous pressure (*CVP*) and central venous oxygen saturation (*ScvO*_*2*_). Resuscitation with intravenous fluids, vasopressors, and packed red blood cells is titrated to specific end-points, including CVP of 8 to 12 mm Hg, mean arterial pressure (*MAP*) of at least 65 mm Hg, and ScvO_2_ of at least 70 %. Inotropic therapy is recommended in patients with low cardiac output despite adequate volume and MAP. Recent controlled clinical trials have challenged the efficacy of this approach for reducing mortality among patients with septic shock. *HCT* hematocrit

Guidelines from the SSC published in 2013 also recommend goal-directed resuscitation during the first 6 hours of septic shock (Fig. 2). In this approach, treatment with intravenous fluids is titrated to specific endpoints, including CVP of 8 to 12 mm Hg and ScvO_2_ of at least 70 %. The SSC recommends placing a central venous catheter to monitor these variables and using a minimum of 30 ml per kg of fluids during initial resuscitation. Other goals of resuscitation include the use of vasopressor therapy to achieve an MAP of at least 65 mm Hg in patients with refractory hypotension as well as inotropic therapy in patients with low cardiac output. In patients with ScvO_2_ persistently below 70 % during the first 6 hours, the SSC advocates the use of packed red blood cell transfusions with a target hematocrit of at least 30 %.

These recommendations are based primarily on evidence from the 2001 trial by Rivers and colleagues, which showed that EGDT reduced the absolute risk of in-hospital mortality by 16 %. Dozens of reports since the trial by Rivers and colleagues have shown improved patient outcomes with EGDT [25–46]. However, the majority of these were non-randomized studies and thus prone to selection bias and confounding variables [47]. Furthermore, the bundled approach of EGDT precludes identification of which elements of the protocol are primarily responsible for reductions in mortality reported in clinical trials.

## Goals of early goal-directed therapy: central venous pressure and central venous oxygen saturation

Recently, three large randomized controlled trials enrolling a total of 4,183 patients were completed to re-examine the effect of early goal-directed resuscitation on outcomes in patients with septic shock: the Protocolized Care for Early Septic Shock (ProCESS) trial in the United States, the Australasian Resuscitation in Sepsis Evaluation (ARISE) trial, and the Protocolised Management of Sepsis (ProMISe) trial in England (Table 2). Each trial used inclusion criteria similar to the original study by Rivers and colleagues and was powered to detect a 6 % to 8 % absolute mortality reduction [48].

**Table 2:** Randomized trials of early goal-directed therapy for patients with septic shock

Trial	Study setting	Sample size	Baseline characteristics of patients receiving EGDT			EGDT in the first 6 hours				Mortality
			APACHE II score	Lactate, mmol/l	Source of sepsis	Total fluids, l	Vasopressor therapy, %	Red cell transfusion, %	Inotropic therapy, %	EGDT vs. usual care, %
Rivers et al. [7]	Single center in USA	263	21.4 ± 6.9	7.7 ± 4.7	38.5 % lungs, 25.6 % urinary, 35.9 % other	4.9 ± 2.9	27.4	64.1	13.7	44.3 vs. 56.9^a^ (*P* = 0.03)
ProCESS [49]	31 centers in USA	1,341	20.8 ± 8.1	4.8 ± 3.1	31.9 % lungs, 22.8 % urinary, 45.3 % other	2.8 ± 1.9	54.9	14.4	8.0	21.0 vs. 18.9^a^ (*P* = 0.83)
ARISE [50]	51 centers in Australia and New Zealand	1,591	15.4 ± 6.5	6.7 ± 3.3	36.5 % lungs, 18.7 % urinary, 44.8 % other	2.5 ± 1.2	66.6	13.6	15.4	18.6 vs. 18.8^b^ (*P* = 0.90)
ProMISe [52]	56 centers in England	1,251	18.7 ± 7.1	7.0 ± 3.5	36.5 % lungs, 17.3 urinary, 46.2 % other	2.2 ± 1.4	53.3	8.8	18.1	29.5 vs. 29.2^b^ (*P* = 0.90)

The Protocolized Care for Early Septic Shock (*ProCESS*), Australasian Resuscitation in Sepsis Evaluation (*ARISE*), and Protocolised Management of Sepsis (*ProMISe*) trials failed to replicate positive findings of the original trial by Rivers and colleagues [7]. The study by Rivers and colleagues was conducted at a single emergency department in a low-income community of Detroit, Michigan. It had a high control group mortality rate, which likely reflects health problems unique to an impoverished patient population as well as delays in treatment. Still, a subgroup analysis in the ARISE trial showed that early goal-directed therapy (*EGDT*) did not improve mortality in patients with increased disease severity (Acute Physiology and Chronic Health Evaluation II (*APACHE II*) score >25, n = 69). Control group mortality rates were markedly lower in the ProCESS, ARISE, and ProMISe trials, which may reflect broad shifts in clinical practice over the last decade toward earlier initiation of antibiotics and vasopressor therapy as well as conservative thresholds for blood transfusion. Indeed, the ARISE trial reported a median time of 70 minutes between initial presentation and administration of antibiotics in the EGDT group versus 67 minutes in usual care

^a^Mortality at 60 days

^b^Mortality at 90 days

The ProCESS trial enrolled patients drawn from 31 academic hospitals in the United States who were diagnosed with septic shock [49]. Patients were randomly assigned to one of three treatment groups: EGDT with continuous monitoring of CVP and ScvO_2_, protocolized standard therapy that did not require continuous monitoring, and usual care. In the usual care group, patient care was directed by clinicians acting without a standardized protocol. Patients in the three groups received significantly different volumes of intravenous fluids within the first 6 hours and those in the EGDT group were most likely to receive vasopressors, inotropes, and red cell transfusions. Despite these differences, no significant change in 60-day mortality or need for organ support was identified.

The ARISE trial in Australia and New Zealand offered concordant evidence [50]. Management of sepsis in Australasia does not typically incorporate goal-directed resuscitation protocols advocated by the SSC. Apart from antimicrobial therapy and source control, standard care during the first 6 hours comprises less aggressive fluid infusions and earlier use of vasopressors than what is recommended by SSC guidelines [51]. The ARISE study enrolled patients drawn from 51 urban and rural hospitals who were diagnosed with septic shock. Patients were randomly assigned to receive either EGDT or usual care. Measurement of CVP and ScvO_2_ using central lines was strictly limited to EGDT and was not permitted in the usual care group. Patients in the EGDT group received significantly more fluids and were more likely to receive vasopressors, inotropes, and red cell transfusions compared with the usual care group. However, no significant difference in 90-day mortality, need for organ support, or length of hospital stay was identified.

The ProMISe trial showed findings consistent with evidence from ProCESS and ARISE and also reported on cost-effectiveness of the EGDT protocol [52]. Patients with septic shock were drawn from 56 hospitals in England and were randomly assigned to receive either a 6-hour sepsis care bundle with EGDT or usual care that did not incorporate continuous monitoring of CVP and ScvO_2_. Patients in the EGDT group received significantly more fluids and were more likely to receive vasopressors, inotropes, and red cell transfusions than those in usual care. However, no significant difference in 90-day mortality was found. Compared with those receiving usual care, patients in the EGDT group had significantly higher organ failure scores at 6 hours and longer stays in the ICU and were more likely to require advanced cardiovascular support. Investigators also showed that average costs in the EGDT group were higher than in usual care but this difference was not statistically significant.

The ProCESS, ARISE, and ProMISe trials reported control group mortality rates that were markedly lower compared with the original trial by Rivers and colleagues. This likely reflects gradual improvements in intensive care since the 1990s, including the adoption of SSC guidelines that support early identification of sepsis and prompt initiation of antibiotics [53]. Indeed, patients assigned to usual care received fluid boluses within the first 6 hours and antibiotics prior to randomization. Titrating fluids to CVP and ScvO_2_, however, did not confer survival benefit to most patients. It remained unclear whether a subset of patients who fail to respond to initial resuscitation may benefit from such measures. The recent trials of EGDT also did not address whether monitoring CVP and ScvO_2_ is beneficial during the initiation of mechanical ventilation in patients with septic shock, when the risk of acute cardiovascular collapse is increased.

## Goals of early goal-directed therapy: mean arterial pressure

In patients with hypotension despite adequate fluid repletion (defined as CVP of at least 8 to 12 mm Hg) or evidence of cardiogenic pulmonary edema, the SSC recommends the use of vasopressors to maintain blood pressure. The benefit of vasopressors in patients with refractory septic shock is well supported by evidence from randomized controlled trials [54]. The SSC currently advocates an MAP goal of 65 mm Hg during the first 6 hours of treatment. Owing to the theoretical risk of coronary ischemia and acute renal and hepatic failure, higher blood pressure goals are not advised, except for patients with atherosclerosis or chronic hypertension [55]. This recommendation is based on evidence from observational studies and one small randomized trial by Bourgoin and colleagues [56–60], which showed no differences in tissue oxygenation or mortality for patients who received higher versus lower MAP targets.

In 2014, a randomized controlled trial called Sepsis and Mean Arterial Pressure (SEPSISPAM) was conducted at 29 centers in France to re-examine high versus low MAP goals in patients with septic shock [61]. The trial also sought to determine whether a subgroup of patients with chronic hypertension benefited from treatment with an MAP target of 80 to 85 mm Hg. Investigators found no significant difference in mortality between those treated with a low versus high MAP target. They also found that the incidence of atrial fibrillation was significantly increased among those in the high-target group compared with the low-target group (6.7 % versus 2.8 %, *P* = 0.02). Investigators showed that chronic hypertension patients who were treated with a higher MAP target were significantly less likely to require renal replacement therapy but did not have increased survival compared with the low-target group. These findings support an MAP goal of 65 mm Hg in most patients with septic shock and suggest that a higher MAP goal may decrease morbidity among those with chronic hypertension.

The optimal timing of vasopressors relative to fluid infusion remains more controversial. Recently, a large multi-center observational study was undertaken in Canada, the United States, and Saudi Arabia to address this question [62]. In a retrospective analysis of 2,849 patients with septic shock, investigators found that mortality was lowest when vasopressors were delayed by 1 hour and infused from hours 1 to 6 following onset of shock. These findings are consistent with those of other retrospective cohort studies that support the early initiation of vasopressors [63, 64]. This approach may decrease the volume of fluids necessary to maintain blood pressure. Indeed, patients in the recent ProCESS, ARISE, and ProMISe trials were more likely to receive vasopressors and required less fluids than patients in the original study by Rivers and colleagues (Table 2). Still, prospective randomized trials are needed to elucidate the optimal timing of vasopressors and volume of fluids during initial resuscitation.

## Goals of early goal-directed therapy: hemoglobin concentration

In the original trial by Rivers and colleagues, packed red blood cell transfusions were used as part of the EGDT protocol and subsequently became one of the key components of sepsis care bundles advocated by the SSC. The SSC recommends using blood transfusions during the first 6 hours of septic shock, but guidelines for when to initiate therapy remain ambiguous and suggest that most patients are eligible for a liberal transfusion threshold. Goals of transfusion include a hematocrit of at least 30 % or a hemoglobin concentration of between 7.0 and 9.0 g/dl. This recommendation is based on evidence from the Transfusion Requirements in Critical Care (TRICC) trial conducted in 1999 [65]. The TRICC trial was a randomized study that enrolled 838 critically ill patients, among whom only 16.7 % had septic shock. Investigators showed that a liberal transfusion threshold of 10.0 to 12.0 g/dl did not significantly improve mortality compared with a conservative threshold of 7.0 to 9.0 g/dl.

Recently, the Transfusion Requirements in Septic Shock (TRISS) trial re-examined transfusion thresholds in patients with sepsis [66]. This randomized study enrolled 998 patients from 32 ICUs in Scandinavia and showed that a hemoglobin threshold for transfusion of 7.0 g/dl resulted in similar rates of mortality, ischemic events, and use of life support in comparison with a threshold of 9.0 g/dl. The conservative approach also decreased the use of blood products by 50 %. In the ProCESS, ARISE, and ProMISe trials, patients receiving EGDT were transfused according to goals described in the original trial by Rivers and colleagues: a target hematocrit of at least 30 % if ScvO_2_ remained below 70 % despite appropriate fluid resuscitation. As a result, patients receiving EGDT were about twice as likely to receive transfusions in comparison with those receiving usual care but this did not improve mortality. These studies collectively suggest that a conservative transfusion threshold of 7.0 g/dl is safe for the majority of patients with septic shock.

## Alternatives to central venous pressure and central venous oxygen saturation

One of the key challenges of treating shock is the measurement of intravascular volume and oxygen delivery during fluid bolus therapy. This is of particular importance in patients with sepsis, who are at high risk of fluid overload. Previous studies have estimated that only 50 % of hemodynamically unstable patients are fluid-responsive, highlighting the need for accurate measurement of volume status [67, 68]. Physical examination and chest radiography are simple methods of assessing volume but are statistically insensitive and unreliable. Static measurements of CVP and pulmonary capillary wedge pressure can be used but also have low sensitivity and specificity for predicting fluid responsiveness [11, 69]. This is likely due to the nonlinear relationship between volume and pressure, which results from variable compliance of the cardiovascular system. The use of ScvO_2_ as an indirect marker of cardiac output and tissue oxygenation is also controversial. Previous studies have shown that ScvO_2_ does not always approximate mixed venous oxygen saturation (SvO_2_), a more accurate marker of total body oxygen consumption [70].

**Fig. 3:**
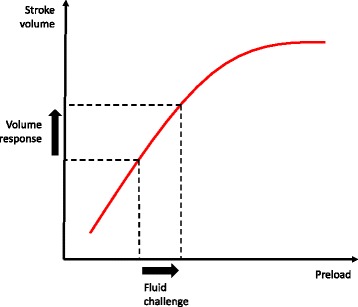
Measuring fluid responsiveness by cardiac ultrasound. A patient is considered fluid-responsive if left ventricular function falls along the ascending portion of the Frank-Starling curve. Additional fluids given above this zone do not increase cardiac output and worsen the risk of volume overload. Measurement of stroke volume and cardiac output by Doppler ultrasound may allow more accurate estimation of fluid responsiveness in patients receiving intravenous fluid therapy

This has prompted the development of alternative methods of monitoring fluid responsiveness in the ICU. Dynamic indices such as variation in stroke volume, pulse pressure, and the diameter of inferior vena cava (IVC) offer the advantage of real-time assessment of hemodynamic status. However, measurement of stroke volume and pulse pressure variation is typically reserved for patients who are receiving mechanical ventilation [71, 72]. The IVC diameter can be measured in spontaneously breathing patients by ultrasound but provides only an indirect assessment of CVP [73]. Other techniques such as the passive leg-raise maneuver and mini-fluid challenge have thus been proposed [74, 75]. The mini-fluid challenge requires a bolus of 100 to 500 ml of crystalloids infused over 10 to 30 minutes, whereas the passive leg-raise acts as a ‘self’ fluid challenge that increases cardiac preload. In these techniques, an ultrasound cardiac output monitor is used to estimate a patient’s fluid responsiveness from position on the Frank-Starling curve. A patient is considered fluid-responsive if cardiac output increases by at least 10 % to 15 % after the fluid challenge (Fig. 3).

Several ongoing trials are investigating the potential role of real-time monitoring of cardiac output to guide fluid resuscitation in sepsis. A Goal-Oriented Non-Invasive Sepsis Trial (AGONIST) is a randomized study in Singapore that will investigate the passive leg-raise maneuver and ultrasound cardiac output monitor for patients with sepsis. The Bioimpedance Analysis in Septic Intensive Care Unit Patients (BIOVISION) is testing another noninvasive method of volume assessment. Bioimpedance analysis has been used for several decades to measure volume status and guide fluid management in patients receiving hemodialysis [76]. In this technique, a small alternating current is passed between electrodes on the hand and foot. The measured drop in voltage reflects resistance to electrical flow or ‘impedance’, which is inversely proportional to total body water.

The role of microcirculation in shock and resuscitation is another potential area of future research. Tissue perfusion and oxygenation are commonly assessed via measurements of arterial lactate concentration. However, previous randomized trials of lactate-guided resuscitation have offered conflicting evidence of survival benefit, and the SSC does not currently advocate a specific goal for lactate reduction during initial treatment of septic shock [77, 78]. Direct observation of microcirculation may offer more accurate assessments of vital organ blood flow and oxygenation [79, 80]. Unfortunately, measurement of microvascular flow is limited to easily accessible organs such as the skin and tongue, and there is considerable inter-operator variation with currently available techniques.

## Emerging paradigms in sepsis trials

In a subset analysis of the ARISE trial, investigators showed that EGDT did not improve mortality in patients with increased severity of illness [50]. The quantification of disease severity in critically ill patients, however, remains a significant challenge in clinical trials and may confound comparative analyses of treatment effect. Scoring systems such as the Acute Physiology and Chronic Health Evaluation are commonly used but were not originally designed for stratifying patients in clinical trials and have been validated in only the first 24 to 48 hours of hospital admission. These population-based statistical models predict ICU mortality but do not accurately reflect pathology in individual patients [81]. Disease severity would likely be better characterized by using molecular diagnostics. Unfortunately, whereas dozens of sepsis biomarkers have been proposed, none has demonstrated sufficient specificity and sensitivity for clinical use [82]. Recent studies suggest that gene expression profiling might offer an alternative approach to stratifying patients in clinical trials [83].

Another barrier to evidence-based sepsis care arises from limitations inherent in traditional randomized controlled trials, which are ill equipped for answering clinical questions in patients with evolving disease states and myriad treatment interventions. Dynamic ‘adaptive’ trial designs have recently emerged as an alternative for studies in critical care. As data accumulate in an adaptive trial, the allocation ratio is changed to favor the treatment group that appears superior. This approach allows investigators to achieve statistical significance with smaller samples and test multiple treatment options that are modifiable as disease severity changes among participants [84]. Adaptive clinical trials have been used previously in many oncology studies and may offer greater safety and efficiency than classic randomized trials [85].

## Conclusions

Conventional management that focuses on early antibiotics and targeted resuscitation has contributed to improvements in survival of patients with septic shock over the last decade. New evidence from the ProCESS, ARISE and PRoMISe trials, however, suggests that structured ‘early goal-directed resuscitation’ with routine placement of a central venous catheter, monitoring of mixed venous oxygen saturation and aggressive red cell transfusion does not improve outcomes in most patients with septic shock. The nuances of fluid and vasopressor administration in early septic shock remain incompletely defined. Further, development and validation of practical methods for accurately assessing optimal fluid administration is needed. Future studies that seek to address these issues will likely benefit from emerging novel techniques, including molecular diagnostics and adaptive trial designs.

## Abbreviations

ARISE: Australasian Resuscitation in Sepsis Evaluation
CVP: Central venous pressure
EGDT: Early goal-directed therapy
ICU: Intensive care unit
IVC: Inferior vena cava
MAP: Mean arterial pressure
ProCESS: Protocolized Care for Early Septic Shock
ProMISe: Protocolised Management of Sepsis
ScvO_2_: Central venous oxygen saturation
SSC: Surviving Sepsis Campaign
TRICC: Transfusion Requirements in Critical Care
